# Supplementary material for: Plasma metabolome study reveals metabolic changes induced by pharmacological castration and testosterone supplementation in healthy young men
Source: Sci Rep. 2022 Sep 23;12:15931. doi: 10.1038/s41598-022-19494-w (PMC9508133; doi:10.1038/s41598-022-19494-w)
Supplement: Supplementary file 1 — Supplementary Figures. [file 41598_2022_19494_MOESM1_ESM.docx]

**Supplementary Data**

**Plasma metabolome study reveals metabolic changes induced by pharmacological castration and testosterone supplementation in healthy young men**

**Jéssica de Siqueira Guedes^1, 2^; Indira Pla^3^; K. Barbara Sahlin^3^; Gustavo Monnerat^1,5^; Roger Appelqvist^3^; György Marko-Varga^3,6^; Aleksander Giwercman^4,7^; Gilberto Barbosa Domont^2^; Aniel Sanchez^3*Ω^; Fábio César Sousa Nogueira^1,2*Ω^; Johan Malm^3,4Ω^**

^1^ Laboratory of Proteomics, LADETEC, Institute of Chemistry, Federal University of Rio de Janeiro, Rio de Janeiro, 21941-598, Brazil

^2^ Proteomics Unit, Institute of Chemistry, Federal University of Rio de Janeiro, Rio de Janeiro, 21941-909, Brazil

^3^Clinical Protein Science & Imaging, Biomedical Centre, Department of Biomedical Engineering, Lund University, BMC D13, 221 84 Lund, Sweden

^4^ Section for Clinical Chemistry, Department of Translational Medicine, Lund University, Skåne University Hospital Malmö, 205 02 Malmö, Sweden

^5^ National Institute of Cardiology, Rio de Janeiro, 22240-006, Brazil

^6^ First Department of Surgery, Tokyo Medical University, 6-7-1 Nishishinjiku Shinjiku-ku, Shinjuku-ku, Tokyo, 160-0023, Japan

^7^ Molecular Reproductive Medicine, Department of Translational Medicine, Lund University, Malmö, 214 28, Sweden

**Corresponding author:* [*fabiocsn@iq.ufrj.br*](about:blank)*;* [*aniel.sanchez@med.lu.se*](mailto:aniel.sanchez@med.lu.se)

**^Ω^** *Considered last authors*

**
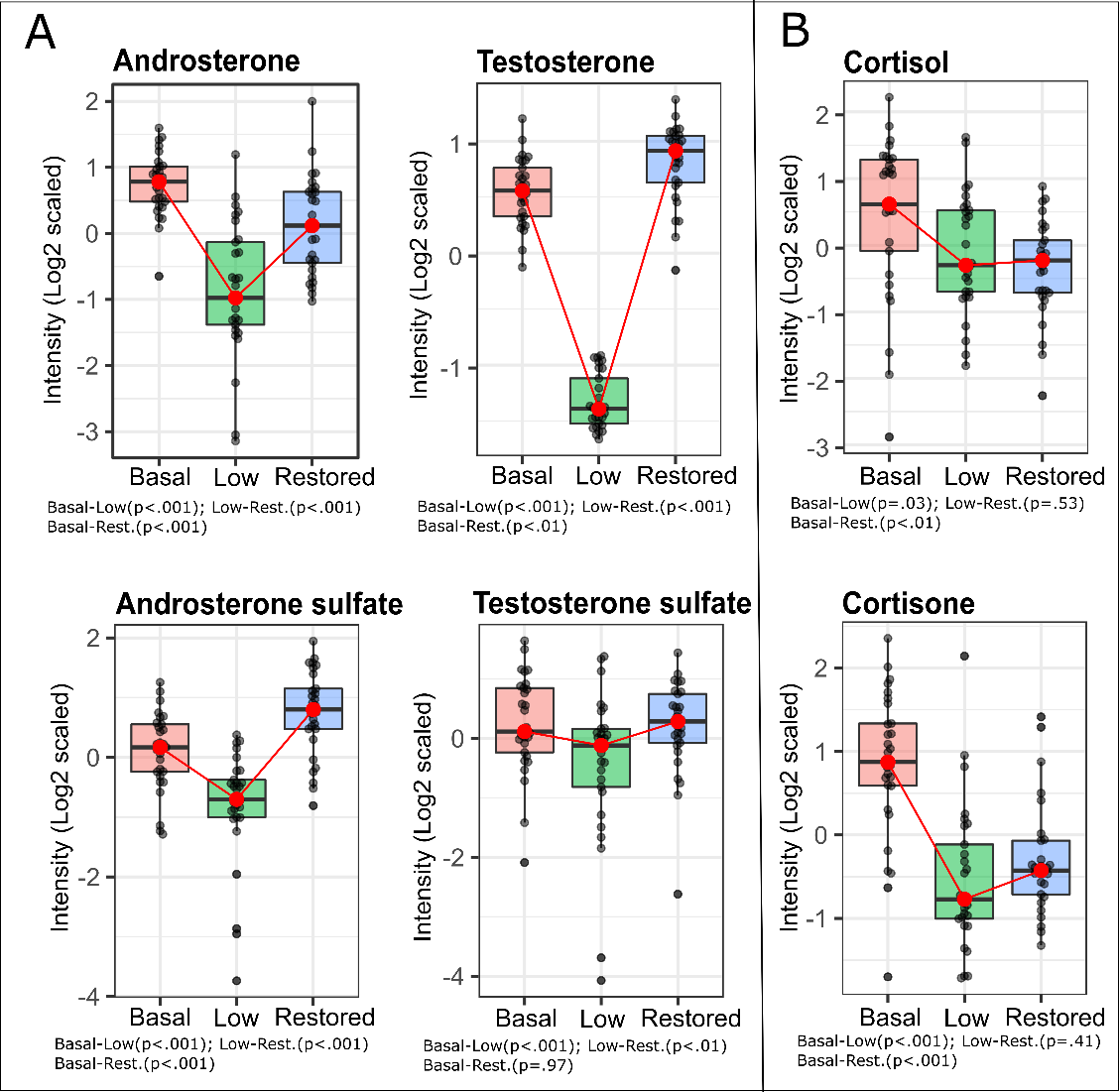
**

**Figure S1.** Steroids' expression after TD and TS. A) Levels of testosterone and its metabolites. B) Levels of cortisol and cortisone. Boxplot colors: Pink, basal testosterone group; Green, low testosterone group; Blue, restored testosterone group.

**­­­**
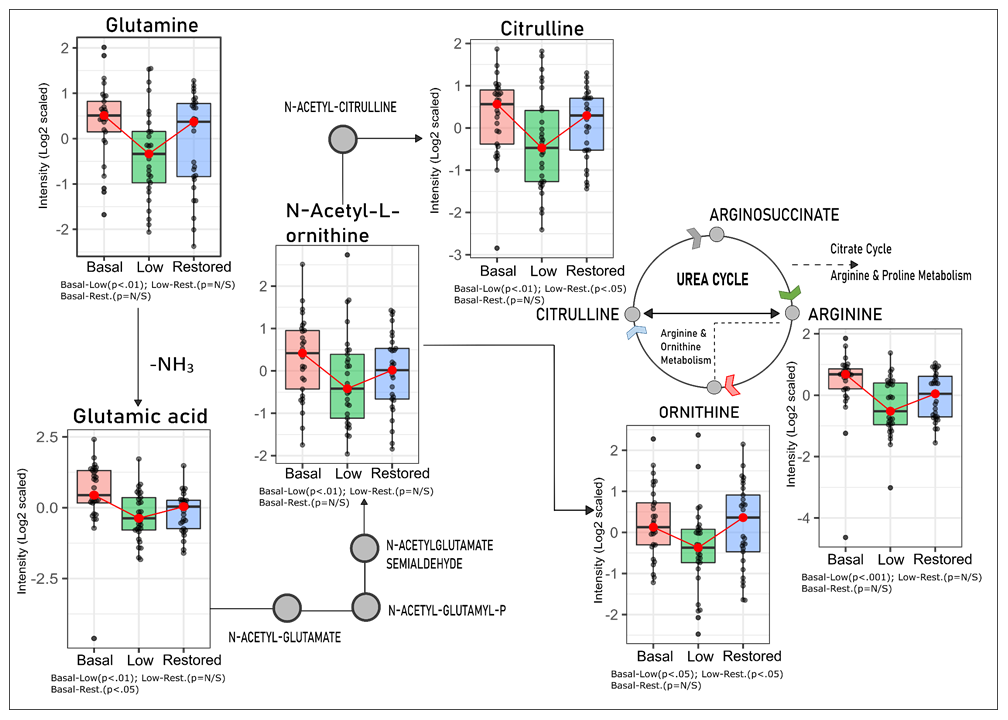


**Figure S2. Testosterone and Arginine Biosynthesis.** Intermediate compounds of arginine biosynthesis present low expression after the TD. Ornithine and Citrulline were restored after the testosterone replacement (*p*-value <0.05, comparison between Low and Restored group). *N*-Acetylornithine, Glutamine, and Arginine tended to restore based on median values (not statistically significant, comparison between Low and Restored group). Boxplot colors: Pink, basal testosterone group; Green, low testosterone group; Blue, restored testosterone group.
